# Rapid, Safe, and Tolerable Healing of Pediatric Skin Injuries with Blue Light Therapy: An Observational Case Series

**DOI:** 10.3390/children12060801

**Published:** 2025-06-19

**Authors:** Nicolosi Biagio, Bastarelli Eleonora, Gregorini Mirco, Ciprandi Guido

**Affiliations:** 1 Complex Injuries and Burn Center, Department of Health Professions, Meyer Children’s Hospital, 50139 Florence, Italy; 2 Wound Care Team, Outsourcing Consumption Appropriateness, Azienda USL Toscana Centro, 50139 Florence, Italy; 3 Department of Health Professions, Meyer Children’s Hospital, 50139 Florence, Italy; 4 Pediatric and Plastic Surgeon, Sapienza University, 00185 Rome, Italy; 5 Wound Care, Catholic University of the Sacred Heart, 00168 Rome, Italy

**Keywords:** blue light, incontinence-associated dermatitis, medical device-related pressure injuries, moisture-associated skin damage, phobiomodulation, pressure injuries

## Abstract

Background/Objectives: Pediatric skin injuries represent a clinical challenge, especially in cases with complex etiology or with severe pain. Blue light is an emerging technology with potential application in pediatric wound care. The aim of this case series was to describe the clinical use of blue light in pediatric patients with injuries of varying etiologies and mechanisms, and to assess its long-term effectiveness and sustainability in treatment. Methods: Twelve hospitalized pediatric patients (0–12 years old) with skin injuries were included in this case series. The etiologies of wounds treated with photobiomodulation were incontinence-associated dermatitis, peristomal injuries, and pressure injuries. The injuries were assessed using specific classification tools and pain scales. The wounds were treated every three days using a medical device that emits blue light (410–430 nm). When necessary, wounds were also treated with appropriate dressings. Results: All injuries responded positively to photobiomodulation therapy and showed a rapid resolution of inflammation. Complete wound resolution was achieved in 11 of 12 cases. The average healing time was 3.7 days. Four injuries achieved resolution with only one application of blue light. Scores from the pain perception scales showed that the blue light treatment was well tolerated by pediatric patients, showing high parental compliance. No side effects or adverse events were observed. Conclusions: Blue light photobiomodulation proved to be a safe, well-tolerated, and effective technology in the treatment of pediatric skin injuries, with good acceptance by young patients and families. More structured clinical trials would be needed to validate the efficacy of blue light in pediatric injuries.

## 1. Introduction

In neonatal and pediatric care, skin injuries represent a complex challenge for the professionals involved because they require specific preparation, advanced skills, and gentle, rapid, and minimally invasive approaches. Effective management of wounds in infants and children involves adequate knowledge of the causes and risk factors associated with different types of injuries.

Most dressings and guidelines for prevention and treatment are derived from studies and practices on adults, despite anatomical and physiological differences; in many cases, pediatric wound care specialists must adapt products for use in children [1].

Infant skin differs from adult skin in structural and functional terms, and although anatomically mature, it is a more delicate and vulnerable structure [2].

To reduce morbidity and mortality, it is essential to maintain skin integrity and achieve a timely diagnosis of chronic wounds.

This need is particularly relevant in pediatrics, as the skin of newborns and children has structural and functional characteristics that differ from that of adults. Specifically, the stratum corneum is thinner, less cohesive, and its barrier function is still developing, especially in premature infants. Capillary density is higher, but the vessels are more fragile, exposing the skin to a greater risk of pressure-related damage and friction or shear forces [3,4].

Moreover, the epidermis and dermis in children are less tightly bound, making the skin more vulnerable to injury even from minimal mechanical stress. Thermoregulation is less efficient, and the body surface area relative to weight is greater, which increases heat dispersion and fluid loss in the presence of open wounds [3]. From an immunological perspective, the inflammatory response may be less regulated and more prone to imbalance, leading to a higher risk of secondary infections.

Recent data indicate that in pediatric intensive care settings, skin injuries—particularly pressure injuries (PIs) and medical device-related pressure injuries (MDRPIs)—are associated with a significant increase in morbidity, including pain, infection risk, longer hospital stays, and the need for specialist interventions [5,6]. Some studies report that skin complications can also impact neonatal mortality, especially when associated with sepsis, fluid and electrolyte imbalance, or delayed wound healing [3,4].

These anatomical and physiological characteristics not only increase the risk of developing conditions such as pressure injuries, peristomal moisture-associated skin damage (pMASD), and incontinence-associated dermatitis (IAD), but also pose significant challenges in wound management, especially when pain or procedural discomfort is involved.

One of the goals of integrated wound management in pediatric subjects is to minimize pain and reduce emotional distress for both patients and parents.

In recent years, photobiomodulation with blue light has shown an important impact on healing chronic wounds of different etiologies in adults [7,8,9,10,11]. Blue light acts through the reduction of inflammation and stimulation of microcirculation, promoting tissue regeneration and pain reduction. Photobiomodulation interacts with the tissue’s endogenous chromophores, triggering reactions that lead to the activation of the cellular pathways functional for tissue repair [12,13,14]. Although photobiomodulation for the treatment of pediatric skin injuries is still poorly documented, blue light therapy involves contactless treatment and is neither invasive nor painful, so it could be a viable treatment opportunity for pediatric patients.

This case series aims to describe the clinical experience of using blue light therapy in the routine care of pediatric patients with skin injuries, exploring its potential efficacy and sustainability in different clinical settings.

## 2. Materials and Methods

The study was conducted in accordance with the principles of the Declaration of Helsinki. Parents or legal guardians of the patients provided written informed consent, including authorization for the publication of clinical images.

The patients included in this case series were treated or hospitalized at Meyer Children’s Hospital, Florence (Italy). All patients were treated by pediatric nursing staff experienced in wound care, who also collected data on the wounds treated with blue light. Twelve pediatric patients (0–12 years old) with skin injuries of various etiologies suitable for blue light therapy were included. Patients with known photosensitivity or conditions that could induce photosensitive reactions were excluded. The etiologies of the wounds treated with photobiomodulation included IAD (4), p-MASD (4), PIs (1), and MDRPIs (3).

Pediatric skin wounds were assessed using validated classification tools and age-appropriate pain scales. IAD was classified using the GLOBIAD (Ghent Global IAD Categorization Tool), which categorizes lesions into four stages based on erythema (1) and skin loss (2), with (A) or without infection (B) [15]. Peristomal injuries were assessed using SACS 2.0 (Skin Alteration Classification System), designed to evaluate localization, severity, and type of peristomal skin damage [16].

To identify superficial wound infections, the NERDS (non-healing, exudative, redness, debris, smell) criteria were applied [17], while pressure injuries were staged according to the EPUAP classification (European Pressure Ulcer Advisory Panel) [18].

Pain was assessed using validated scales appropriate to the patient’s age: the N-PASS (Neonatal Pain, Agitation, and Sedation Scale) for neonates (0–1 month) [19], the FLACC (Face, Legs, Activity, Cry, Consolability) scale for infants (1 month–1 year) [20], and the Wong–Baker FACES Pain Rating Scale for children aged 7 years and older [21].

### Intervention

All wounds in this case series were treated with blue light photobiomodulation using the EmoLED device (Emoled, srl—Florence, Italy), a CE-marked phototherapy medical device indicated for skin injuries in individuals over 16 years of age. The treatment consisted of blue light irradiation (410–430 nm; 120 mW/cm^2^) for 60 or 120 s, applied to the wound surface after cleansing and before dressing, with sessions performed at intervals of 3 to 4 days. The device, classified as a non-contact, noninvasive Class IIa medical device, was held at a distance of 40 ± 10 mm from the wound. The recommended protocol involved two treatments per week. In the context of this study, the use of the device was considered off-label, as is often the case for medical devices in pediatric populations.

In addition to photobiomodulation, wound management involved the use of dressings selected according to the wound characteristics. The most frequently used products included DACC-coated gauzes for wounds with infection risk, gelling fiber dressings for highly exudative wounds, thin foam dressings for fragile or post-surgical skin, and silicone contact layers for superficial injuries or skin exposed to friction. Dressing selection was based on wound type, exudate level, anatomical site, and patient age, in accordance with best clinical practices and pediatric skin care principles. The frequency of dressing changes varied according to wound characteristics and exudate level but was generally performed every 3 to 4 days in most cases, in line with standard pediatric wound care practices. For IAD, dressings were typically changed at each diaper change, while for peristomal MASD, dressing changes coincided with the replacement of the collection device. In other cases, such as pressure injuries or MDRPIs, dressings were replaced every 2 to 3 days, depending on exudate and wound progression.

The number of blue light sessions performed per patient, along with dressing types and wound outcomes, is summarized in Table 1.

## 3. Results

The following cases were selected to discuss in further detail as they were representative of the different etiologies treated with blue light therapy in this case series. These cases include examples of IAD, p-MASD, and MDRPI, thereby illustrating the clinical variability and therapeutic application of blue light across a heterogeneous pediatric wound population.

The wounds examined in this case series and their clinical characteristics are listed in Table 1. Eight males and four females were treated; four patients were newborns less than 1 month old, three patients were infants between 4 and 7 months old, and the remaining patients were older than 9 years old (9–12).

Complete wound resolution was achieved in 91.6 percent of cases (11 of 12), while partial healing was found in an infected p-MASD with surgical wound complication who had only two treatments with photobiomodulation but still showed significant clinical improvement.

The average healing time was 3.7 days, with an average of 1.4 blue light treatments. IADs and p-MASDs healed with only 1 to 2 blue light treatments, while 2 to 4 applications were needed for PIs.

Table 2 shows the evaluation of perceived pain assessed in all cases according to different scales, depending on the age of the child. In the case of the four newborns (aged less than 1 month), the N-PASS was applied. For the three infants (1 month–1 year old), the FLACC was applied. For the five children over 7 years of age, the Wong–Baker FACES Pain Rating Scale was used.

The three newborns showed signs of mild agitation, characterized by increased motor activity, moderately elevated muscle tone and, in one case, changes in vital signs. One of the infants (37 + 2 weeks) showed neither pain nor agitation, with a total N-PASS score of zero. The scores obtained described conditions of minimal discomfort, with no evidence of pain. Only mild agitation was manifested in infants, with no evidence of pain. The scores indicated discomfort, characterized by mild restlessness, but there was no need for analgesic intervention. Older children reported no pain, and facial descriptions indicated slight differences in expression, attributable to moderate agitation or restlessness, but with no evidence of distress.

None of the patients presented with neurological deficits that could have interfered with pain perception or response during treatment.

No analgesic medications were administered during the procedures, as the observed pain scores did not indicate the need for pharmacological pain relief.

No side effects or adverse events were observed during and after all light treatments.

Below is a detailed description of four cases that provides an understanding of the different clinical conditions faced and the therapeutic strategies applied.

### 3.1. Case 1

**Figure 1:**
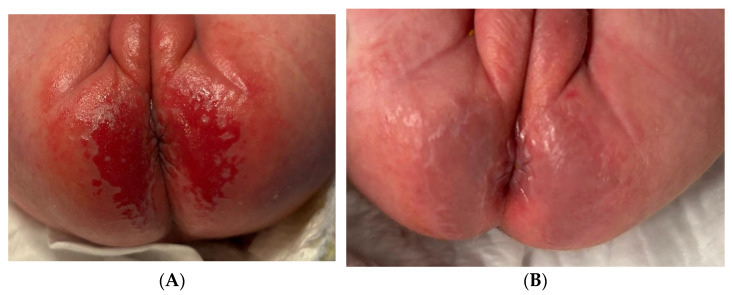
GLOBIAD grade 2A IAD in newborn. (**A**) Initial lesion; (**B**) healing after 5 days.

This female newborn (33 + 1 weeks) had GLOBIAD grade 2A IAD (Figure 1). Observable clinical features included intense, well-demarcated erythema in the gluteal and perianal folds, erosive-type injuries with loss of integrity with symmetrical distribution, typical of contact dermatitis associated with incontinence, often caused by prolonged exposure to urine and feces. Two blue light photobiomodulation treatments were performed on the entire lesion area, spaced three days apart, with each session lasting 120 s. No additional therapies were used on the area; only a care strategy was performed, which included frequent diaper changes (hourly) and hygiene with disposable wipes and pH-neutral detergent without alcohol or perfume. Healing was achieved in 5 days. The lesion had clinical features indicating good healing: significant reduction of erythema, restoration of skin integrity, and disappearance of erosive injuries. The skin surface appeared smooth and well moisturized, with no signs of scaling.

### 3.2. Case 2

**Figure 2:**
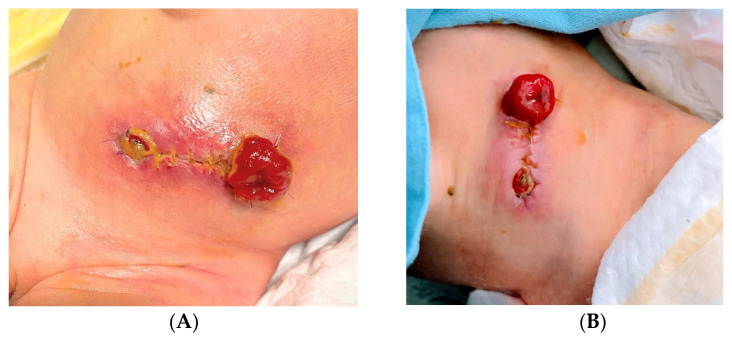
pMASD in abdominal ostomy with SACS 2.0 T5. (**A**) Initial lesion; (**B**) lesion after 6 days.

The second case described is a pMASD in abdominal ostomy with SACS 2.0 T5-L3 in a 34-week-old gestational age (GA) newborn with anorectal malformation (ARM) (Figure 2). The patient was a male and presented with local infection at the peristomal site, consistent with NERDS (non-healing, exudative, redness and bleeding, debris, smell) criteria, indicative of critical bacterial colonization of the surgical wound. The stoma was clearly visible, with a longitudinal surgical wound still awaiting healing and intense perilesional erythema extending to the surrounding area. Under the pouching system, a DACC (dialkylcarbamoylchloride) gauze was applied as the first dressing, placed directly on the peristomal skin, covered with a transparent breathable film, to provide a protective barrier against contaminants and maintain adherence of the collection system. Before each packing of the ostomy pouching system, application of photobiomodulation with blue light was performed for 60 s each. After 6 days and two blue light treatments, the intense erythema was resolved, and the surgical wound was healing, with reduced signs of inflammation and critical colonization. Given the rapid course of recovery, the patient was discharged with appropriate parental training in collection system management; the patient was evaluated at the next follow-up, in which blue light therapy was no longer necessary.

### 3.3. Case 3

**Figure 3:**
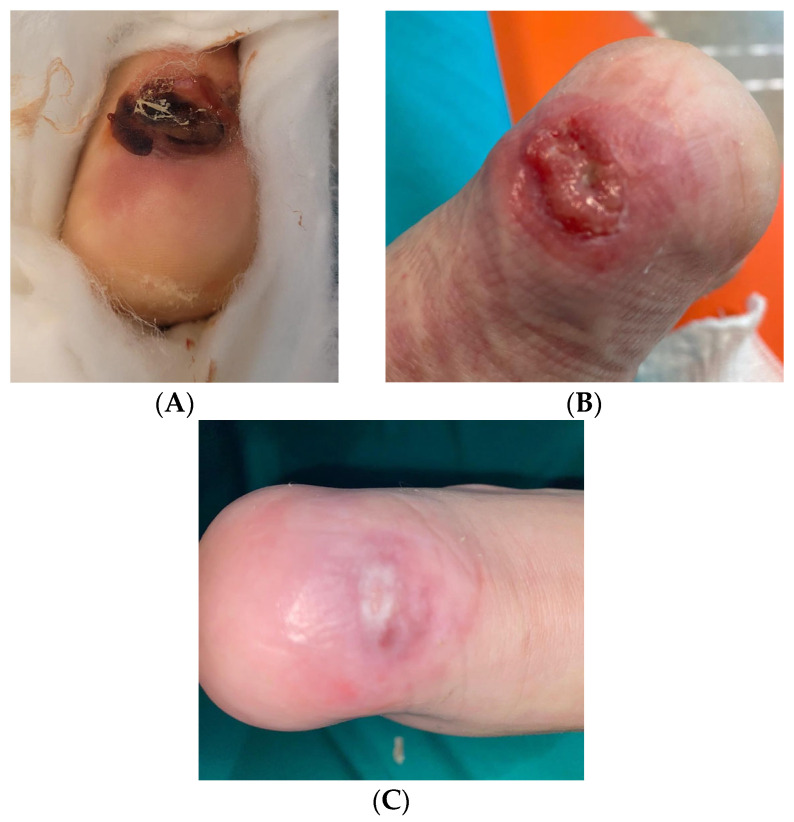
MDRPI on the calcaneus formed after the cast removal. (**A**) Initial lesion; (**B**) ulcerative area with hypergranulation tissue; (**C**) healing after 13 days.

This case described an MDRPI on the calcaneus of a 9-year-old male child, formed after the removal of a cast (Figure 3). The lesion presented with a dark-colored central necrotic area that was unstageable. An erythematous area (a clear sign of inflammation) was visible around the necrotic area. Incorrect treatment performed at home overstimulated the proliferative phase of healing, consequent to an excessively moist environment, an excess of topical product and of the pressure load on the lesion. The ulcerative area with hypergranulation tissue was bright red in color and shiny and friable in appearance.

Treatment was started with DACC gauze, kept in place with an elastic compression bandage. At each dressing change, blue light treatment was performed a total of 4 times for 120 s each. The ulcer improved significantly, with obvious reduction of hypergranulation tissue and no signs of inflammation such as peripheral erythema or edema, indicating a resolution of the active inflammatory process. Complete healing occurred within 13 days: the previously ulcerated area had completely re-epithelialized, with the formation of new skin tissue of smooth and uniform appearance and a light pink color, indicative of an effective and complication-free process of cell regeneration.

### 3.4. Case 4

**Figure 4:**
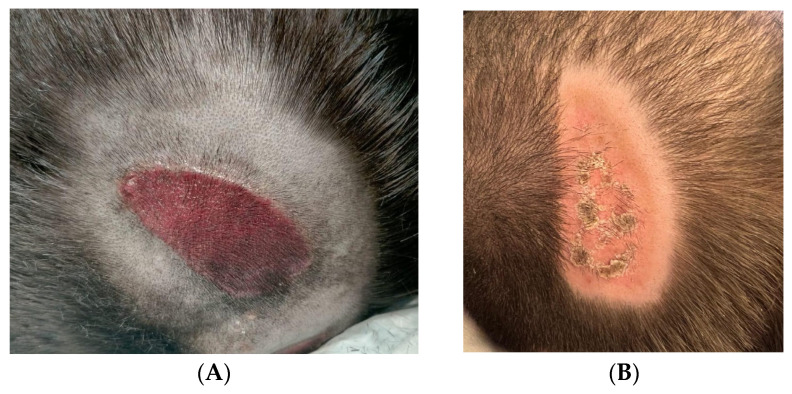
PI in 12-year-old boy hospitalized for respiratory failure and respiratory infection. (**A**) Initial lesion; (**B**) healing after 5 days.

This 12-year-old boy was hospitalized for respiratory failure and respiratory infection and had a lesion resulting from pressure exerted by the strap of a noninvasive ventilation mask (total face) (Figure 4). The lesion had a wide and well-demarcated extent with loss of superficial skin integrity. The skin was completely eroded, with exposure of the dermis (EPUAP stage 2). The staining was deep red, and the edges of the lesion were well defined, suggesting localized pressure trauma. No clear signs of active infection or exudate were present. Treatment was performed with two applications of photobiomodulation with blue light, lasting 120” each. The lesion was simply covered with a silicone layer. Complete healing was achieved in 5 days, with the necessary measures of positioning and promotion of a protected and moist lesion environment.

## 4. Discussion

In recent years, photobiomodulation with blue light has been used in several settings with positive results, producing clear evidence of its efficacy and safety [22].

This therapy has been used by specialized healthcare personnel to treat wounds of various etiologies, particularly of vascular or diabetic origin [23,24,25,26]. Blue light therapy proved to be effective not only in accelerating but also in unlocking the re-epithelialization process of wounds that do not progress despite the application of a proper standard of care [9,27,28,29].

The effectiveness of this therapy has also been evidenced by clinical trials showing an increase in healing speed and probability of chronic venous wounds [7], an improvement in re-epithelialization in sclerosis skin ulcers [10], and the best recovery and advancement of tissue repair towards complete wound healing in pressure ulcers [8].

Phototherapy in infants and children is currently used mainly to resolve neonatal jaundice and treat skin disorders. The use of photobiomodulation, particularly with blue light, in the field of pediatric wound healing is not widely used and validated to date; nevertheless, the safety profile and noninvasive mode of application make this methodology an ideal candidate to be a useful tool in the treatment of childhood ulcers. So, although there are differences between adults and children in skin structure and etiologies of lesion onset, blue light therapy acts on cellular pathways involved in tissue repair that are common in both children and adults, and even in ulcers with different etiologies [29].

One of the key elements shared by the different types of injuries included in this case series—namely pressure injuries (PIs), medical device-related pressure injuries (MDRPIs), and incontinence-associated dermatitis (IAD)—is the central role of localized inflammation in initiating and perpetuating tissue damage [4,5,30,31]. Whether triggered by mechanical pressure or prolonged exposure to moisture and irritants, the inflammatory response leads to cellular stress, disruption of the skin barrier, and delayed healing.

Blue light photobiomodulation has demonstrated the ability to modulate inflammatory pathways by reducing the expression of pro-inflammatory cytokines (such as IL-1β, TNF-α) and reactive oxygen species, while promoting anti-inflammatory mediators and mitochondrial activity [12,13,14,22,29]. This anti-inflammatory effect represents a unifying mechanism of action that supports its beneficial impact across wound types with different etiologies but similar inflammatory pathogenesis. Therefore, the efficacy observed in this series may be partially attributed to this targeted modulation of the inflammatory microenvironment, which allows more rapid progression to tissue repair phases such as proliferation and remodeling [7,8,9,10,27,28,29].

To the best of our knowledge, this work is the first one to evaluate photobiomodulation with LED blue light in pediatric wound care.

Pediatric PIs, including MDRPIs, are a significant issue in pediatric care settings, especially in neonatal and pediatric intensive care units. These injuries can arise due to pressure from devices such as ventilation masks, cannulas, monitoring bands, and other instruments that, when applied continuously or without adequate protection, compromise skin integrity [5]. Children are particularly vulnerable to these injuries because of the characteristics of their skin, which is thinner and more fragile than that of adults [3]. Prevention of pediatric pressure ulcers requires continuous monitoring, appropriate device selection and application, and, when necessary, the use of protective dressings [32].

Standard treatments for PIs primarily include the use of advanced dressings, moisture control, pressure reduction through support devices, and careful management of patient positioning. In the case of MDRPI, the focus is on continuous monitoring of the skin area in contact with the device and frequent repositioning to prevent damage [6]. Regarding healing times, the duration varies depending on the severity of the lesion: Stage I and II injuries can heal within a few days or weeks with prompt and appropriate treatment, while Stage III and IV injuries may take weeks or months, often requiring advanced interventions such as debridement or negative-pressure wound therapy (NPWT). The outcome depends on the timeliness of treatment and the proper management of predisposing conditions [4,33].

IAD in pediatric settings is commonly observed in neonates and young children, particularly in those with prolonged use of diapers or issues with fecal and urinary incontinence. The main cause is prolonged exposure of the skin to moisture, typically from urine and feces. IADs manifest clinically with erythema, edema, erosive injuries and, in some cases, secondary skin infections, such as candidiasis. Standard treatment for IAD focuses on prevention through proper skin hygiene, the use of gentle cleansing products, skin protection with barrier products such as zinc oxide or silicone-based creams, and strategies to keep the skin dry [4,34].

Healing times for IAD are generally rapid, with visible improvement within a few days of starting treatment and complete resolution within one week in milder cases. However, in cases complicated by fungal or bacterial infections, healing times may be significantly prolonged and require specific therapies [4,31].

In our experience, all patients treated with blue light photobiomodulation showed positive results in response to treatment. In 11 out of 12 cases, the injuries achieved full healing with very rapid resolution times. Healing times in this series (average 3.7 days) appear shorter compared to those commonly reported in the literature for pediatric IAD (up to 7 days), p-MASD (5–10 days), and PIs (ranging from a few days to several weeks depending on severity) [30,33]. Thirty-three percent (4) of the injuries achieved resolution with only one application of blue light. One treatment effect we noticed in all cases was the rapid resolution of inflammation, which allowed the injured tissue to proceed with tissue repair more quickly.

Scores from the pain perception scales showed that the blue light treatment was well tolerated by pediatric patients who showed only discomfort or agitation, probably related to the staff manipulation rather than treatment with the medical device. Therefore, the therapy was well accepted by patients, also showing high parental compliance. No adverse events or side effects were observed during the blue light application, confirming the safety already established in adults.

This case series shows limitations such as the small number of patients and the variability of dressings associated with blue light treatment, the absence of a control group, and the lack of randomization, which prevent any direct causal inferences regarding the contribution of blue light alone to the observed healing outcomes. However, the strengths of this therapy were shown to be adaptability to a wide heterogeneity of injuries, absence of side effects, and excellent applicability in pediatric settings.

## 5. Conclusions

Blue light photobiomodulation has proven to be a safe, well-tolerated, and potentially effective technology in the treatment of various pediatric skin injuries, with rapid healing time and good acceptance by young patients and families. So blue light is a promising therapeutic opportunity for pediatric wound care. However, more structured clinical trials with a larger population would be needed to validate the efficacy of blue light photobiomodulation in pediatric wound care.

## Figures and Tables

**Table 1:** The table summarizes the characteristics of pediatric clinical cases by type of skin lesion. For each case, the age and sex of the patients, staging of the lesion, anatomical location, duration of treatment, number of sessions or duration of applications, outcome obtained, and additional notes on the type of dressing used are given.

Case	Age at Treatment	Sex	Stage	Localization	Applications Performed/ Duration	Healing Time (Days)	Outcome	Note
**IAD**								
Esophageal atresia in prematurity **(Case 1)**	33 + 1 weeks GA	F	GLOBIAD 2A	Perineal	2 After 3 days from 1st (60″)	5	H	
Myelomeningocele	37 + 2 weeks GA	M	GLOBIAD 2A	Perineal	1 (120″)	4	H	
Anorectal malformation (ARM) post-recanalization	6 months	F	GLOBIAD 2B (Candida)	Perineal	2 Every 3 days from 1st (120″)	6	H	2nd dressing DACC
Anorectal malformation (ARM) post-recanalization	7 months	F	GLOBIAD 1B	Perineal	1 (120″)	2	H	2nd dressing DACC
**p-MASD**								
Stoma in anorectal malformation (ARM) **(Case 2)**	34 Weeks GA	M	SACS 2.0 T5-L3 with local infection and complicated surgical wound	Peristomal	2 Every 3 days from 1st (60″)	6	PH	Dressing DACC
Stoma in anorectal malformation (ARM)	36 + 2 weeks GA	M	SACS 2.0 T5-L2	Peristomal	1 (60″)	3	H	
Gastrostomy in short bowel syndrome (SBS)	4 months	M	SACS T5-L2	Abdomen	1 (120″)	3	H	Thin foam
Crohn’s disease	12 years	F	SACS T5-L2	Peristomal	2 After 3 days from 1st (120″)	5	H	
**PIs and MDRPIs**								
PI in spastic tetraparesis	8 years	M	Suspected deep damage	Sacrum	2 After 3 days from 1st (120″)	5	H	
MDRPI post-cast removing **(Case 3)**	9 years	M	Outcome of unstageable injury	Heel	4 Every 3 days from 1st (120″)	13	H	Dressing DACC
MDRPI (tracheostomy tube) in polytrauma	11 years	M	3rd EPUAP	Sternum handlebar	3 Every 3 days from 1st (120″)	8	H	Gelling fiber
MDRPI (total face mask) in respiratory failure and infection **(Case 4)**	12 years	M	2nd EPUAP	Occiput	2 Every 3 days from 1st (120″)	5	H	Silicon layer

GA: gestational age. H: healed. PH: partially healed. DACC: dialkylcarbamoylchloride.

**Table 2:** Pain scale scores.

N-PASS						
GA (Weeks + Days)	Facial Expression	Cry Irritability	Extremities Tone	Behavior State	Vital Signs	Total Score
33 + 1	0	1	1	1	0	3
34	0	1	1	0	0	2
36 + 2	0	1	0	1	1	3
37 + 2	0	0	0	0	0	0
**FLACC**						
**Age ** **(months)**	**Face**	**Legs**	**Activity**	**Cry**	**Consolability**	**Total Score**
4	1	0	1	0	0	2
6	0	1	1	0	0	2
7	0	1	0	1	0	2
**Wong–Baker FACES Pain Rating Scale**						
Age (years)	Face description				Wong-Baker score	
8	Smiling face, quiet, no signs of pain				0—No Hurt	
9	Neutral face, quiet, only slight restlessness				0—No Hurt	
11	Face relaxed, no signs of distress				0—No Hurt	
12	Smiling face, slight agitation, but no signs of pain				0—No Hurt	
13	Quiet face, slightly pensive, but no pain				0—No Hurt	

## Data Availability

The original contributions presented in this study are included in the article. Further inquiries can be directed to the corresponding author.
